# Evaluation of liquefaction potential in central Taiwan using random forest method

**DOI:** 10.1038/s41598-024-79127-2

**Published:** 2024-11-11

**Authors:** Chih-Yu Liu, Cheng-Yu Ku, Yu-Jia Chiu, Ting-Yuan Wu

**Affiliations:** Department of Harbor and River Engineering, National Taiwan Ocean University, Keelung, 202301 Taiwan

**Keywords:** Liquefaction, Seismic activity, Artificial neural network, Random forest, Earthquake, Potential, Environmental sciences, Natural hazards, Engineering

## Abstract

Liquefaction is a significant geotechnical hazard in seismically active regions like Taiwan, threatening infrastructure and public safety. Accurate prediction models are essential for assessing soil susceptibility to liquefaction during seismic events. This study evaluates liquefaction potential in central Taiwan using the random forest (RF) method. The RF models were developed with a dataset of 540 soil and seismic parameter sets, including depth, effective and total overburden stresses, SPT-N values, fine soil content, earthquake magnitude, peak ground acceleration, and historical liquefaction occurrences. Rigorous validation techniques, such as cross-validation and comparisons with observed liquefaction events, confirm the RF model’s effectiveness, achieving an accuracy of 98.89%. The model also quantifies predictor importance, revealing that the SPT-N value is the most critical soil factor, while peak ground acceleration is the key seismic factor for liquefaction prediction. Notably, the RF model outperforms simplified procedures in accuracy, even with fewer input factors. Our case studies show that an accuracy of over 95% can still be achieved, highlighting the RF model’s superior performance compared to conventional methods, which struggle to reach similar levels.

## Introduction

Liquefaction, a geotechnical hazard, poses significant risks to infrastructure and communities in regions prone to seismic activity^1–3^. Notable historical events like the 1964 earthquake in Alaska and Niigata (Japan) and the 1999 Izmit earthquake (Turkey) underscore the potential devastation caused by liquefaction in saturated sandy soils^4,5^. In Taiwan, situated within the seismically active Pacific Ring of Fire, the concern over liquefaction-induced hazards is a critical aspect of disaster preparedness and infrastructure resilience^6^. Given the potential catastrophic impact of liquefaction events, accurate prediction and assessment of liquefaction susceptibility are crucial for disaster preparedness, risk mitigation, and urban planning efforts in Taiwan.

Traditional empirical methods for liquefaction assessment often rely on simplified soil parameters and empirical correlations, which may lack accuracy and fail to capture the complex interactions between soil properties, seismic characteristics, and ground response dynamics^7,8^. In the last five decades, the predominant method for assessing soil liquefaction potential has been the simplified procedure^9^, which was formulated through empirical assessments of field observations and data obtained from field and laboratory experiments.

Employing the simplified procedure requires the estimation of two variables, cyclic resistance ratio (CRR) and cyclic stress ratio (CSR), to evaluate soil liquefaction resistance. An efficient approach to evaluating CRR entails retrieving and analyzing undisturbed soil specimens in laboratory settings. Nonetheless, reproducing in situ stress states within the laboratory environment is commonly difficult, and soil samples acquired through conventional drilling methods frequently exhibit excessive disturbance, compromising the reliability of results^10–12^. Consequently, various field tests, such as the shear-wave velocity measurements (Vs), cone penetration test (CPT), and standard penetration test (SPT), are commonly employed to assess liquefaction resistance^13–16^, with SPT and CPT being preferred due to their extensive data sets and established track records^17,18^.

Empirical methods like the Hyperbolic Function (HBF) method and the National Center for Earthquake Engineering Research (NCEER) method rely on multiple assumptions and approximations^19–22^. Traditional experimental or empirical analyses for assessing liquefaction potential can be time-consuming and labor-intensive. Therefore, there is growing interest in utilizing advanced computational techniques, such as machine learning methods, to develop more accurate and robust predictive models for liquefaction potential.

Applying machine learning models for predicting liquefaction potential presents several advantages over traditional empirical methods^23–27^. Various machine learning techniques, including support vector machines (SVM)^28^, artificial neural network (ANN)^29^, Bayesian belief network (BBN)^30,31^, decision trees^32^, random forest (RF)^33^, and logistic regression^34^, have been utilized to analyze soil liquefaction potential. Among these techniques, experimental studies have shown that the decision tree algorithm outperformed others on the dataset^35,36^. Furthermore, the scarcity of studies incorporating comprehensive datasets encompassing both geotechnical properties and historical liquefaction observations has been a persistent challenge due to data collection complexities.

This study evaluates the liquefaction potential in central Taiwan using the random forest (RF) method. The RF models were developed with a dataset of 540 in situ measurements, including soil and seismic parameters such as depth, effective and total stresses, SPT-N value, fine soil content, earthquake magnitude, peak ground acceleration, and historical liquefaction observations. Rigorous validation, including cross-validation and comparison with observed liquefaction events. This research not only contributes to the advancement of predictive modeling techniques in geotechnical engineering but also holds significant implications for enhancing hazard assessment and risk management strategies in Taiwan’s seismic-prone regions.

## Study area and dataset

### Study area

**Fig. 1:**
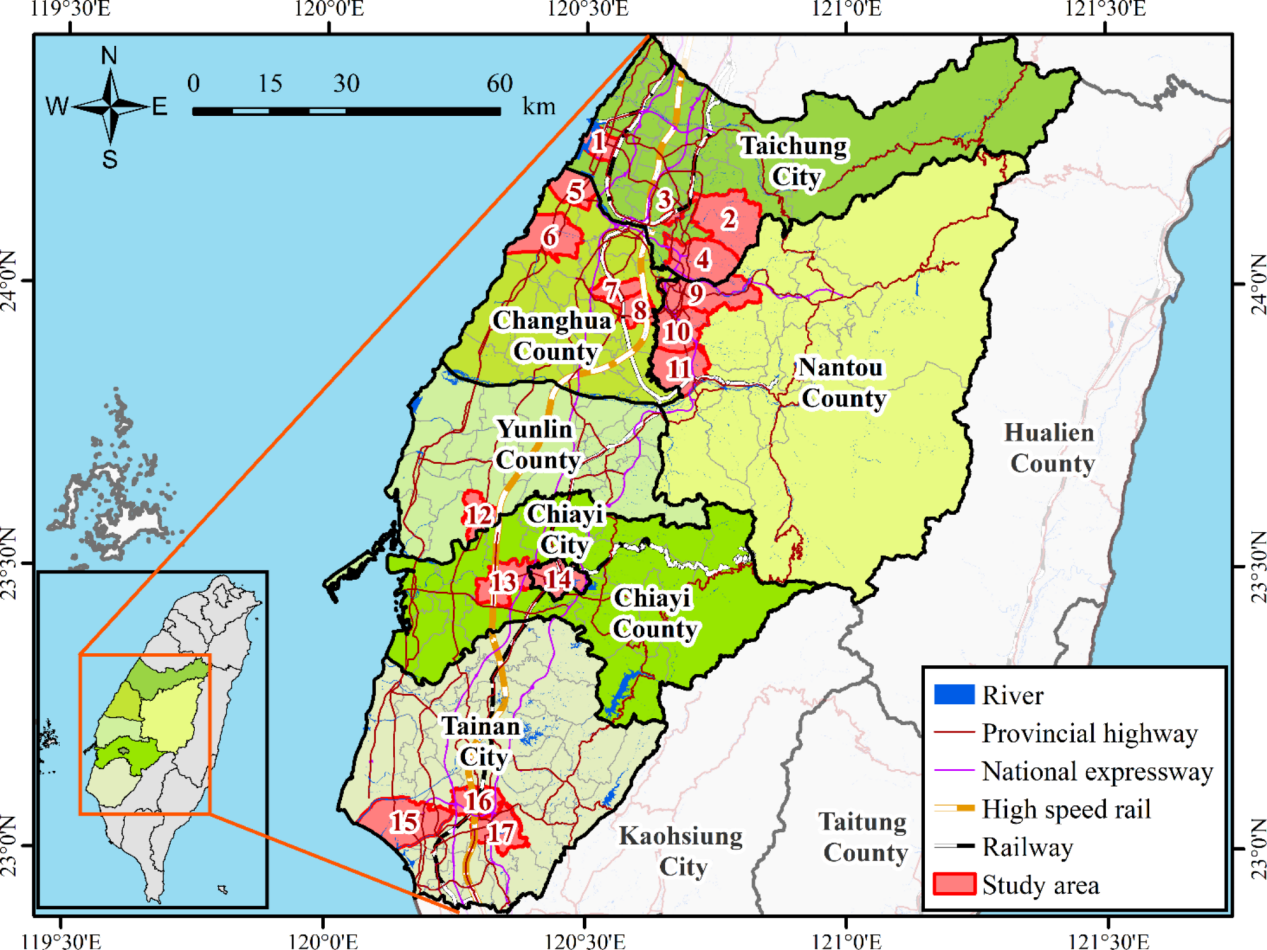
Locations where liquefaction occurred in central Taiwan during the Chi-Chi Earthquake (the figure was produced utilizing ArcGIS 10.8 software).

The seismic event known as the Chi-Chi earthquake struck on September 21, 1999, causing extensive destruction, especially in central Taiwan. This study is focused on the central Taiwan research area, as depicted in Fig. 1, with the locations of liquefaction areas identified during the Chi-Chi Earthquake serving as evidence. Originating along the Chelungpu Fault in western Taiwan, the earthquake traverses the foothills of the Central Mountain Range in Nantou County, revealing the surface exposure of the Chelungpu Fault across a distance of about 105 km. The land neighboring the fault underwent uplift of up to 7 m. In the Wufeng area of Nantou County, central Taiwan, ground accelerations peaked between 0.7 and 1.0 g. The seismic impacts were significant, resulting in tremors felt throughout the entire island, resulting in considerable life loss, residential zones, and extensive damage to infrastructure.

In addition to destructive fault ruptures and numerous fractures, central Taiwan experienced landslides and extensive soil liquefaction, leading to severe disasters. Liquefaction, a phenomenon where soil temporarily loses strength and behaves like a liquid, primarily occurred in specific areas including Taichung City, Changhua County, Nantou County, Yunlin County, Chiayi City, Chiayi County, and Tainan City, as illustrated in Fig. 1. Among these, Taichung City, Changhua County, and Nantou County stand out with significant occurrences of soil liquefaction. Table 1 summarizes the number of soil liquefaction cases in the study area. Taichung City had 69 cases of soil liquefaction and 43 cases of non-liquefaction, while Changhua County reported 111 cases of soil liquefaction and 84 cases of non-liquefaction. In Nantou County, there were 68 cases of soil liquefaction and 4 cases of non-liquefaction.

**Table 1:** The number of soil liquefaction cases in the study area.

No.	Site	County/city	Number of cases		References
			Liquefaction	Non-liquefaction	
1	Wuqi District	Taichung City	33	43	^20^
2	Taiping District	Taichung City	3	0	
3	South District	Taichung City	2	0	
4	Wufeng District	Taichung City	31	0	
5	Shengang Township	Changhua County	25	0	
6	Lukang Township	Changhua County	0	84	
7	Dacun Township	Changhua County	1	0	
8	Yuanlin City	Changhua County	86	0	
9	Caotun Township	Nantou County	5	0	
10	Nantou City	Nantou County	52	3	
11	Mingjian Township	Nantou County	11	1	
12	Beigang Township	Yunlin County	0	43	
13	Taibao City	Chiayi City	0	35	
14	Chiayi City	Chiayi County	0	28	
15	Annan District	Tainan City	11	0	
16	Xinshi District	Tainan City	10	0	
17	Xinhua District	Tainan City	0	2	
18	Others	Others	0	31	^11^
Total			270	270	–

### Dataset

In this investigation, the liquefaction and non-liquefaction database established by Hwang et al.^20^ in 2003 and 2012 post the Chi-Chi Earthquake served as the primary resource. This database, originally developed to study seismic events, was expanded to encompass 540 cases from the 2016 Meinong Earthquake, along with supplementary instances of liquefied and non-liquefied conditions. The study aggregates a comprehensive set of 540 cases, comprising 270 occurrences of soil liquefaction and 239 instances of non-liquefaction, sourced from diverse regions as delineated in Table 1. Furthermore, 31 additional foreign cases retrieved from Japan^11^, categorized as non-liquefaction scenarios, were included to enhance the dataset’s breadth. The dataset analyzed includes a combination of soil and seismic parameters, such as depth, effective and total stresses of overburden, SPT-N value, fine soil content, earthquake magnitude, peak ground acceleration, and the occurrence of liquefaction.

Table 2 presents the descriptions of the datasets used in this study, where factor 1 to factor 7 represent factors pertinent to potential causes of soil liquefaction. This study incorporates various data sources related to potential factors causing soil liquefaction, including depth, effective stresses of overburden, total stresses of overburden, SPT-N value, fine content of soil, magnitude of earthquake, peak ground acceleration, and liquefaction condition (a total of eight factors).

**Fig. 2:**
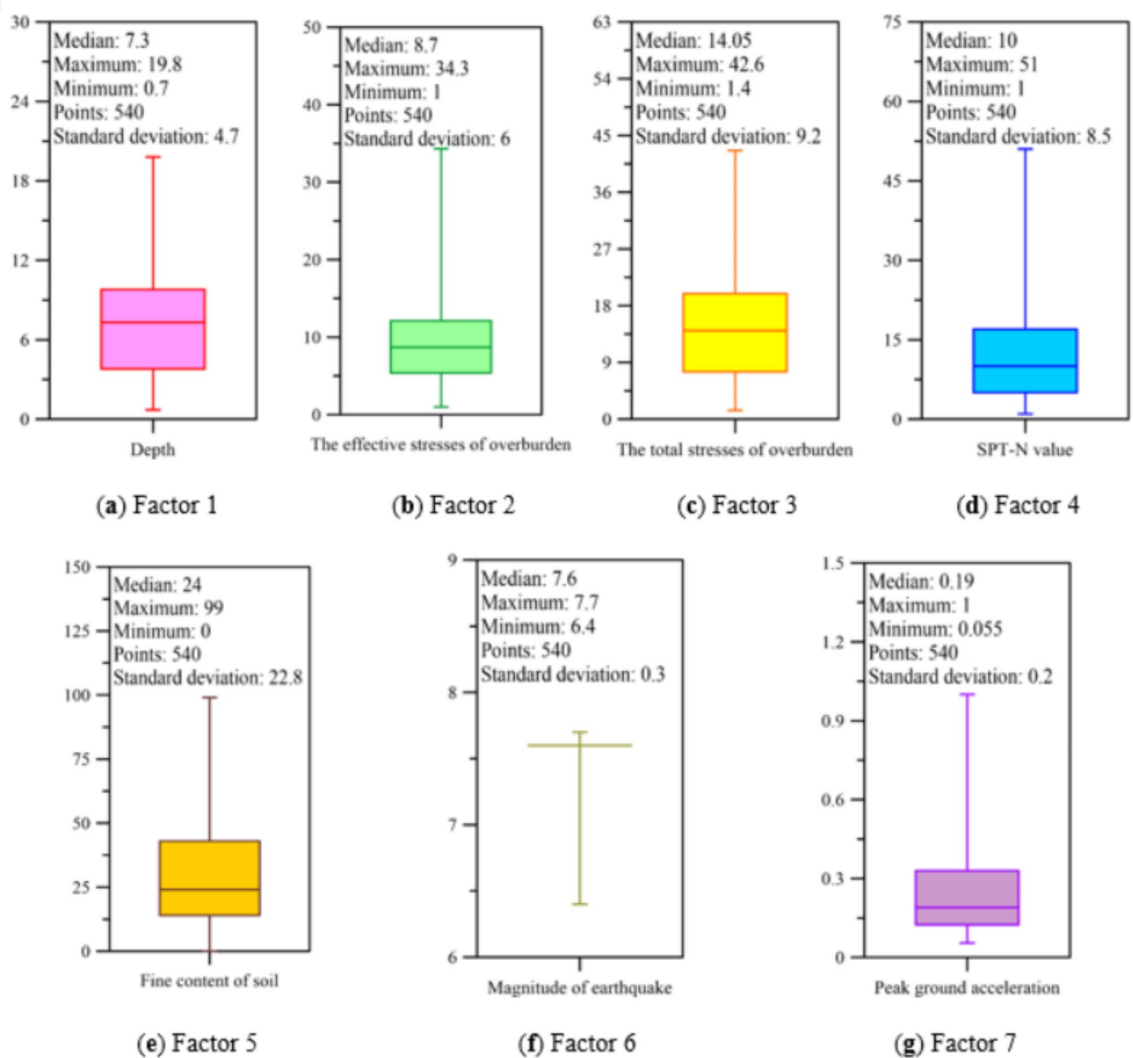
Distribution of input data.

To visualize the distribution of input data, this study employed box plots, a statistical method for analyzing data. As shown in Fig. 2, box plots provide a graphical summary of dataset distribution, capturing essential metrics like the median, quartiles, and potential outliers. In the box plot in Fig. 2, the median, maximum, minimum, and standard deviation are included to illustrate the value distribution of the seven factors.

**Table 2:** Statistical information of the data.

		Factor description	Notation	Unit	Median	Maximum	Minimum	Standard deviation
Soil-related factors	Factor 1	Depth	*Z*	(m)	7.3	19.8	0.7	4.7
	Factor 2	The effective stresses of overburden	${\sigma _v}^\prime$	(kPa)	8.7	34.3	1	6.0
	Factor 3	The total stresses of overburden	${\sigma _v}$	(kPa)	14.05	42.6	1.4	9.2
	Factor 4	SPT-N value	${N_{{\text{60}}}}$	NA	10	51	1	8.5
	Factor 5	Fine content of soil	FC	(%)	24	99	0	2.28
Seismic factors	Factor 6	Magnitude of earthquake	*M*	NA	7.6	7.7	6.4	0.3
	Factor 7	Peak ground acceleration	${A_{{\text{max}}}}$	(g)	0.19	1	0.055	0.2
Liquefaction state	Factor 8	Liquefaction condition	*C*	NA	NA	NA	NA	NA

## Simplified procedure for liquefaction resistance of soils

The simplified method introduced by Seed and Idriss^10^ to assess soil liquefaction susceptibility is a standard process employed to determine the probability of soil liquefaction, a phenomenon marked by the loss of soil strength and its transition into a liquid-like state during seismic occurrences. The primary steps include site investigation, soil classification, evaluation of liquefaction potential, liquefaction analysis, and assessment of liquefaction resistance. Central to the Seed method is the utilization of the CSR, defined as the ratio of cyclic shear stress to confining pressure. This parameter plays a critical role in liquefaction analysis. Seed and Idriss^10^ developed empirical correlations that establish relationships between the CSR, soil properties, and earthquake characteristics. The method then evaluates the liquefaction potential of the soil based on the computed CSR and empirical correlations. Despite its simplicity, it is crucial to acknowledge its reliance on empirical correlations, leading to potential limitations in certain soil and seismic conditions. Throughout the years, the Seed method has been widely adopted and cited in geotechnical engineering literature.

When calculating the evaluation of liquefaction resistance of soils, two variables need to be estimated. The first variable represents the seismic impact on the soil, denoted as the CSR. The second variable indicates the soil’s resistance to liquefaction, expressed as the CRR, also known as the cyclic variable. Seed and Idriss^10^ developed the subsequent equation to compute the CSR:

1 $${\text{CSR}}=0.65 \times \frac{{{A_{\hbox{max} }}}}{g} \times \frac{{{\sigma _v}}}{{\sigma _{v}^{\prime }}} \times {r_d},$$

where ${r_d}$, ${A_{\hbox{max} }}$, ${\sigma _v}$ and ${\sigma _v}^\prime$ are the peak horizontal ground acceleration, stress reduction factor, total and effective stresses, respectively. A plausible approach to assess the CRR involves retrieving and analyzing undisturbed soil samples in laboratory conditions. Various in-situ tests, including the SPT, CPT, and Vs, are commonly employed for liquefaction resistance assessment. Among these, the SPT and CPT are typically preferred due to their extensive databases and historical reliability. The approximation of the clean sand base curve is described by the following equation:

2 $${\text{CR}}{{\text{R}}_{{\text{7}}{\text{0.5}}}}=\frac{1}{{34 - {{{\text{(}}{N_1})}_{60}}}}+\frac{{{{{\text{(}}{N_1})}_{60}}}}{{135}}+\frac{{50}}{{{{\left[ {10{{{\text{(}}{N_1})}_{60CS}}+45} \right]}^2}}} - \frac{1}{{200}},$$

where ${\text{CR}}{{\text{R}}_{7.5}}$ denotes the CRR for magnitude 7.5 earthquakes, ${{\text{(}}{N_1})_{{\text{60}}}}$ denotes the corrected SPT-N value, adjusted for the fine content of the soil. The above equation is only applicable when ${{\text{(}}{N_1})_{60}}<{\text{30}}$. Granular soils with densities too high, ${{\text{(}}{N_1})_{60}} \geq {\text{30}}$, to liquefy are classified as non-liquefiable. This formula is employed in spreadsheet and other routine engineering calculations for approximating clean sand-based analysis techniques. Due to the increase in fines content, CRR increases significantly, whether this increase is due to an increase in liquefaction resistance or a decrease in penetration resistance remains unclear^37^. Therefore, the following equation adjusts ${{\text{(}}{N_1})_{{\text{60}}}}$ to an equivalent clean sand value, ${{\text{(}}{N_1})_{{\text{60}}CS}}$:

3 $${{\text{(}}{N_1})_{60CS}}=\alpha +\beta {{\text{(}}{N_1})_{60}},$$

where coefficients $\alpha$ and $\beta$ are determined based on the following relationships:

4 $$\alpha =\left\{ {\begin{array}{*{20}{l}} {{\text{0, if FC}} \leq {\text{5 \% }}} \\ {{e^{1.76 - (\frac{{190}}{{F{C^2}}})}}{\text{, if 5\% }} \leq {\text{FC}} \leq 3{\text{5\% }}} \\ {{\text{5, if FC}} \geq {\text{35\% }}} \end{array}} \right.,$$

5 $$\beta =\left\{ {\begin{array}{*{20}{l}} {1,{\text{ if FC}} \leq {\text{5 \% }}} \\ {0.99+(\frac{{{\text{F}}{{\text{C}}^{1.5}}}}{{1000}}{\text{), if 5\% }} \leq {\text{FC}} \leq 3{\text{5\% }}} \\ {1.2,{\text{ if FC}} \geq {\text{35\% }}} \end{array}} \right.$$

These equations can be utilized for conventional liquefaction resistance calculations. The back-calculated curve for fines content at 35% closely aligns with the plotted 35% curve.

Seed and Idriss (1982)^12^ introduced correction factors known as magnitude scaling factors (MSF) used for scaling up or down the basic CRR curve, which can be employed to adjust the magnitude of CSR.

6 $${{\text{F}}_{\text{S}}}=\left( {\frac{{{\text{CRR}}}}{{{\text{CS}}{{\text{R}}_{{\text{7}}{\text{0.5}}}}}}} \right){\text{MSF,}}$$

where MSF represents the magnitude scaling factor, ${F_S}$ denotes the factor of safety against liquefaction, and CSR_7.5_ represents the CSR associated with magnitude 7.5 earthquakes.

### National Center for Earthquake Engineering Research (NCEER)

When it comes to assessing soil liquefaction, the NCEER method is widely recognized as a preferred approach for evaluating soil susceptibility during seismic events^9^. The NCEER method typically involves a comprehensive analysis of soil properties, including grain size distribution, density, fines content, and shear strength parameters. Additionally, it considers seismic factors such as peak ground acceleration, distance from the epicenter, and earthquake magnitude. The NCEER method for predicting and determining soil liquefaction potential relies on SPT-N values and fines content (FC) as parameters for predicting liquefaction resistance.

In Taiwan, the SPT is primarily conducted following the American standard ASTM D1586. This standard outlines the testing procedure and provides guidelines for interpreting the results, ensuring that the SPT values obtained in Taiwan are compatible with those from the United States. To elaborate, the ASTM D1586 standard includes specific methods for conducting the test, including equipment specifications, testing procedures, and criteria for evaluating the results. Since Taiwan adheres to this standard, the data collected using SPT in Taiwan can be reliably compared to data obtained elsewhere, particularly in the United States.

The fundamental liquefaction potential analysis based on the NCEER method consists of two primary computations. Firstly, the maximum ground surface acceleration of seismic events is estimated, considering potential or occurring earthquakes in the soil layer. This estimation is used to calculate the CSR using semi-empirical formulas. Secondly, the CRR of the soil layer is estimated based on various investigation and testing data. One of the key features of the NCEER method is its reliance on empirical correlations derived from extensive field observations and laboratory testing. These correlations help quantify the liquefaction potential of soil based on specific soil and seismic parameters.

### Hyperbolic function (HBF) method

The HBF model formulates the CRR using the following equations, which incorporate three parameters, each with unique physical significance^20^:

7 $${\text{CRR}}=A+\frac{{B{{{\text{(}}{N_1})}_{60CS}}}}{{1 - [\frac{{{{{\text{(}}{N_1})}_{60CS}}}}{C}]}},$$

where *A* represents the model parameter corresponding to the inherent liquefaction resistance, typically ranging from 0.06 to 0.10, *B* represents the rate of CRR increase concerning ${{\text{(}}{N_1})_{{\text{60}}CS}}$, typically falling between 0.003 and 0.005, and *C* represents the upper limit of ${{\text{(}}{N_1})_{{\text{60}}CS}}$, ranging from 25 to 45. The three model parameters *A*, *B*, *C*, were quantified as 0.08, 0.0035, and 39, respectively.

### Revised HBF method

Regarding the revised HBF model, the recommended coefficients are defined as follows: *A* = 0.07, *B* = 0.0042, and *C* = 42^20^. The revised HBF model is expressed as:

8 $${\text{CS}}{{\text{R}}_{{\text{7}}{\text{0.5}}}}=0.65 \times \frac{{{A_{\hbox{max} }}}}{g} \times \frac{{{\sigma _v}}}{{\sigma _{v}^{\prime }}} \times \frac{{{r_d}}}{{{\text{MSF}}}},$$

9 $${\text{CRR}}={\text{0}}{\text{0.07}}+\frac{{{\text{0}}{\text{0.0042(}}{N_1}{)_{60CS}}}}{{1 - [\frac{{{{{\text{(}}{N_1})}_{60CS}}}}{{{\text{42}}}}]}}.$$

The revised HBF model, primarily derived based on the reference state of CSR_7.5_ for magnitude 7.5 earthquakes, is used to evaluate CSR_7.5_ and CRR, as well as liquefaction potential.

## **Random forest for liquefaction potential assessment**

**Fig. 3:**
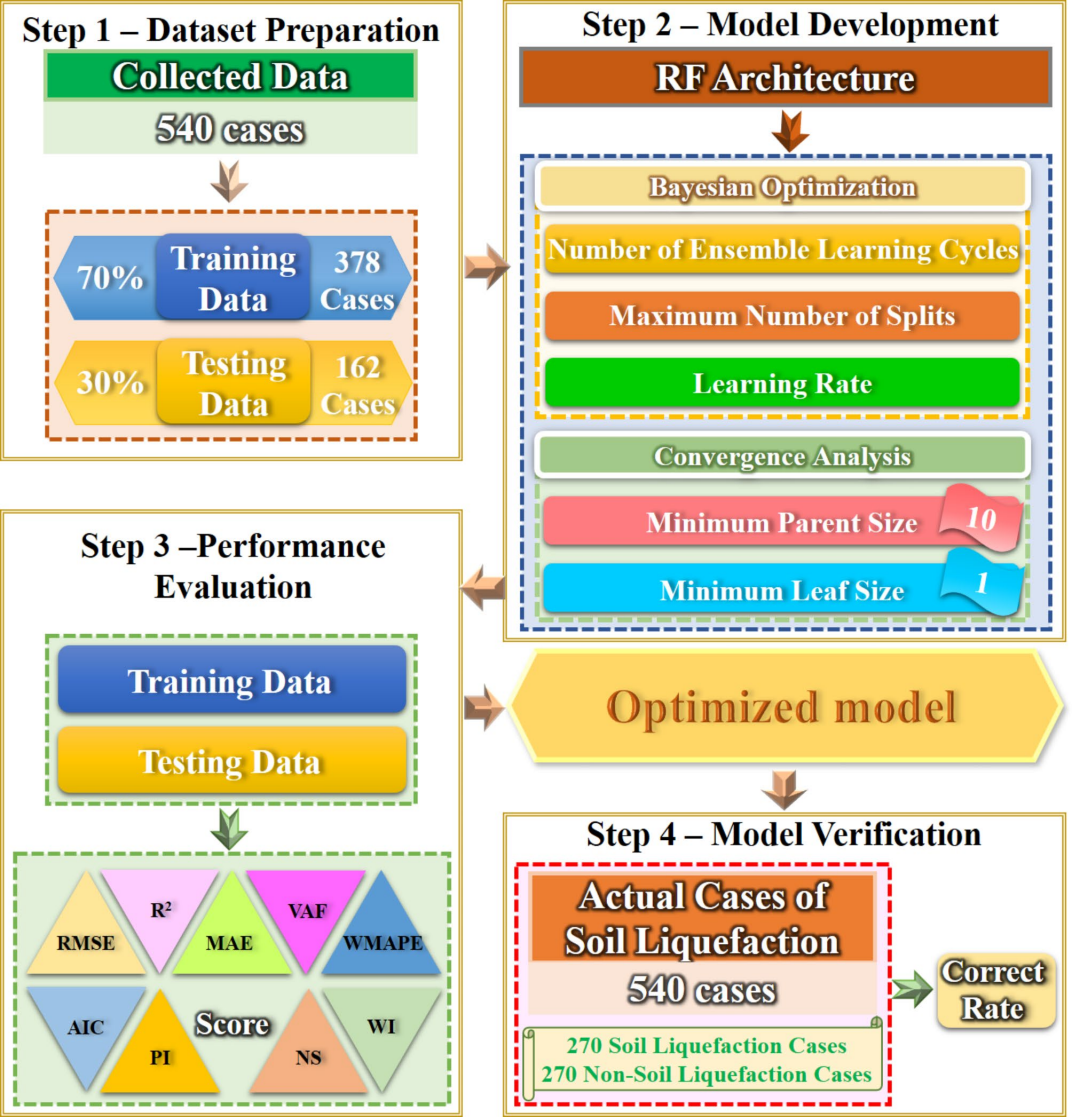
The diagram illustrating the random forest approach for assessing liquefaction potential.

This study utilizes a RF model in machine learning to conduct liquefaction sensitivity analysis in central Taiwan. The procedure of using the RF approach for assessing liquefaction potential is shown in Fig. 3. The key steps include data collection and preparation, feature selection, model construction, hyperparameter tuning, model evaluation, model validation and testing, and interpretation and analysis. The key steps are as follows:

1. Data Collection: This study collects a comprehensive dataset containing features (e.g., soil properties, seismic factors) and corresponding labels (liquefaction occurrence or non-occurrence). This study utilizes both soil-related and seismic factors as data for training and testing. The dataset comprises 540 data points, each including both soil and seismic factors. The data is split into 70% (378 data points) for training and the remaining 30% (162 data points) for testing. The input data forms the basis for training each decision tree within the RF. In this study, random sampling was used to create the training set. We chose a 7:3 ratios for training and testing because this split is widely recognized as optimal for all algorithms, providing a balanced approach to training the model effectively and evaluating its performance^33,38,39^. With 70% allocated to the training set, the model has ample data for learning, while the 30% allocated to testing ensures a sufficiently large dataset to accurately assess the model’s accuracy.

In this study, the decision to use 70% of the total data for training the model is primarily based on two reasons: balancing training and testing data volumes and avoiding overfitting. According to previous research references^40,41^, using 70% of the total data for training can help achieve a balance between the training and testing datasets:

A sufficiently large training set is necessary for the model to learn patterns and rules within the data, while a robust testing set is crucial for accurate model evaluation. Utilizing 70% of the data provides an ample number of samples for training, ensuring that the model can capture the complexities of the data. The remaining 30% is used for testing, allowing the model to be evaluated on a sufficient number of samples. If the testing set is too small, the model’s performance evaluation may lack stability and reliability.

Furthermore, choosing to allocate 70% of the total data for training the model may effectively prevent overfitting. If too much data is assigned to the training set while the testing set remains too small (for instance, using 85% for training and 15% for testing), this can result in overfitting, where the model excels on the training set but performs poorly on the testing set. On the other hand, allocating insufficient data to the training set may hinder the model’s ability to learn essential features, leading to inadequate performance. The 70/30 ratio is widely recognized in the literature as it provides a balanced approach between training and testing, allowing the model to achieve strong generalization capability—enabling reliable predictions on new data.

(2) Out-of-bag (OOB) predictor importance: The OOB samples in RF are employed to evaluate the significance of each feature. This involves training the RF model and using the OOB samples to estimate the reduction in prediction accuracy when a particular feature is permuted.
(3) Model construction: The RF model is initialized with a specified number of decision trees. The RF model is then trained on the training dataset, allowing each tree to grow using a bootstrap sample of the data and selecting the best split at each node based on a subset of features.
(4) Hyperparameter tuning: Key hyperparameters such as the number of trees, maximum tree depth, and the number of features considered for splitting at each node are identified. The convergence of the model performance with respect to different hyperparameters is analyzed to ensure robustness.
(5) Model evaluation: In this study, the RF model undergoes evaluation using a range of performance metrics. These metrics include the coefficient of determination (R²), Nash-Sutcliffe efficiency (NS), variance accounted for (VAF), root mean square error (RMSE), weighted index (WI), Akaike information criterion (AIC), weighted mean absolute percentage error (WMAPE), prediction interval (PI), and maximum absolute error (MAE). The combination of these metrics is used to calculate scores and verify the model’s accuracy. The definitions for the aforementioned performance metrics are outlined as follows.

Firstly, R^2^ is utilized to assess the goodness of fit of the regression model, with values ranging from 0 to 1. The closer the value is to 1, the better the model fits. Equation (10) presents the mathematical expression:

10 $${{\text{R}}^{\text{2}}}={\left( {\sum\limits_{{i=1}}^{T} {({d_i} - {d_{avg}})({y_i} - {y_{avg}})/\sqrt {\sum\limits_{{i=1}}^{T} {{{({d_i} - {d_{avg}})}^2}} } \sqrt {\sum\limits_{{i=1}}^{T} {{{({y_i} - {y_{avg}})}^2}} } } } \right)^2},$$

where *T* is the total number of data points, ${d_{avg}}$ is the mean of the observed values of the dependent variable, ${y_{avg}}$ is the mean of the predicted values of the dependent variable,${d_i}$ is the observed values of the dependent variable, and ${y_i}$ is the predicted value of the dependent variable.

NS is a metric employed to evaluate the precision of models, comparing observed and simulated values to gauge the residual variance relative to measured data variance. A score of 1 denotes flawless model performance, whereas scores below 0 indicate poor performance. Equation (11) depicts the mathematical formulation:

11 $${\text{NS}}=1 - \sum\limits_{{i=1}}^{T} {{{\left( {{d_i} - {y_i}} \right)}^2}/\sum\limits_{{i=1}}^{T} {{{\left( {{d_i} - {d_{avg}}} \right)}^2}} } .$$

VAF measures the proportion of variance in the dependent variable that is explained by the independent variables in the model, as shown in Eq. (12).

12 $${\text{VAF}}=\left[ {1 - \frac{{{\text{SSE}}}}{{{\text{SST}}}}} \right] \times 100{\text{,}}$$

where SSE is the sum of squared errors, and SST is the total sum of squares. Higher values of VAF indicate that a greater proportion of the variability in the dependent variable is accounted for by the model’s independent variables.

RMSE quantifies the disparities between predicted values from a model and actual observations. It represents the square root of the mean of the squared differences between predicted and observed values, as depicted in Eq. (13).

13 $${\text{RMSE}}=\sqrt {\frac{{\text{1}}}{T}\sum\limits_{{i=1}}^{T} {{{\left( {{d_i} - {y_i}} \right)}^2}} } ,$$

WI is a composite measure that integrates various indicators or variables by assigning specific weights to each. Through computation of a unified score, it delineates the relative significance of different factors, as demonstrated in Eq. (14).

14 $${\text{WI}}={\text{1}} - \left[ {\sum\limits_{{i=1}}^{T} {{{\left( {{d_i} - {y_i}} \right)}^2}/\sum\limits_{{i=1}}^{T} {{{\left( {\left| {{y_i} - {d_{avg}}} \right|+\left| {{d_i} - {d_{avg}}} \right|} \right)}^2}} } } \right].$$

AIC is a statistical measure used to evaluate how well a model fits the data while considering its complexity. It strikes a balance between the model’s fit and its simplicity. Lower AIC values indicate a better balance between fit and complexity, indicating a more optimal model. The mathematical expression is illustrated in Eq. (15).

15 $${\text{AIC}}=T \times \ln {\text{(RMSE}}{{\text{ }}^2}{\text{)}}+2k.$$

where *k* is the number of parameters in the model.

The WMAPE evaluates the average percentage error between predicted and observed values, as described in Eq. (16).

16 $${\text{WMAPE}}=\sum\limits_{{i=1}}^{T} {\left| {{y_i} - {d_i}} \right|/\sum\limits_{{i=1}}^{T} {{y_i}} } .$$

PI in statistics is a range of values that is calculated to contain the future outcome of a variable with a specified level of confidence, as described in Eq. (17).

17 $${\text{PI}}={{{\hat {R}}}^{{2}}}+{{(}}0.01 \times {\text{VAF)}} - {\text{RMSE}},$$

where ${{{\hat {R}}}^{\text{2}}}$ is the adjusted R^2^.

MAE is a metric used to evaluate the accuracy of predictions or forecasts. MAE quantifies the magnitude of the largest single error in absolute terms. The equation for MAE can be expressed as:

18 $${\text{MAE}}=\hbox{max} (\left| {{y_i} - {d_i}} \right|),$$

where max denotes the maximum function, which finds the largest absolute difference across all predictions and actual values.

(6) Model Validation: This study employed cross-validation to validate the performance of the model across various subsets of the data. The reliability of the predictions is assessed using the performance metrics to ensure robust and accurate predictions for liquefaction susceptibility. To validate the accuracy of the predicted results, this study also computes the accuracy, which indicates the proportion of correctly predicted instances to the total number of instances in a dataset. Mathematically, this is formulated as:

19 $${\text{Accuracy}}=\frac{{{I_C}}}{{{I_T}}} \times 100\% ,$$

where ${I_C}$ denotes the number of correctly predicted instances, and ${I_T}$ denotes the total number of instances. This formula calculates the ratio of correctly predicted instances to the total number of instances in the dataset.

### Out-of-bag predictor importance

OOB predictor importance is a technique used to assess the importance of features in a RF model. It consists of multiple decision trees, each constructed from different training subsets and feature subsets. Each decision tree is built using different random samples and feature subsets. Due to this randomness, some data points may never be used during training, and these data points are referred to as OOB. OOB predictor importance assesses the contribution of each feature to the model based on its performance in OOB testing. The importance of a feature is calculated based on the number of times the feature is used across all trees and the reduction in average testing error when the feature is used in each tree. OOB predictor importance provides an intuitive way to evaluate the impact of features on the predictive performance of a RF model and helps in selecting the most important features for modeling purposes. We incorporated OOB predictor importance to pinpoint influential predictors within the specific model being utilized.

**Fig. 4:**
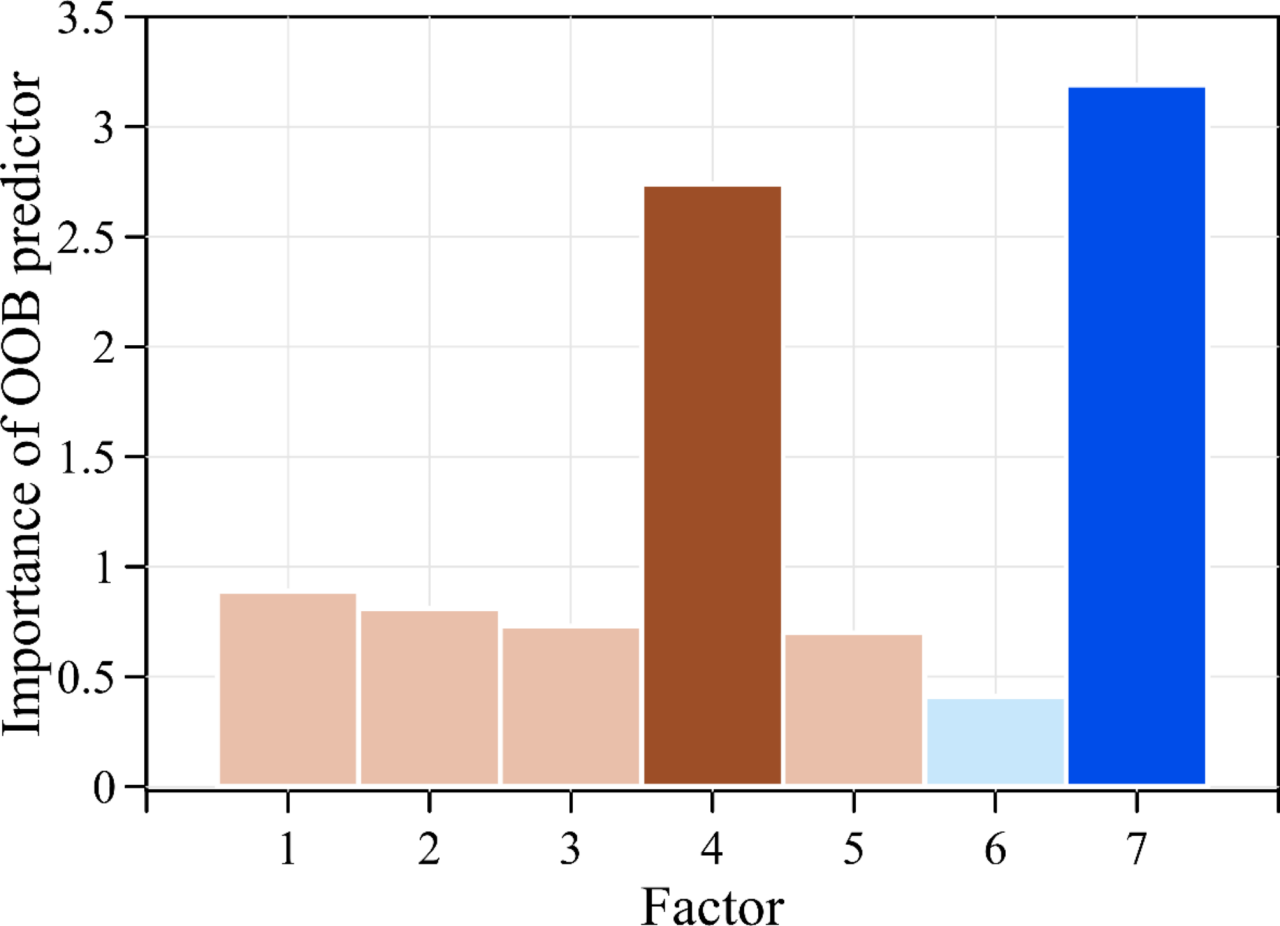
Variable importance plot using the OOB predictor.

Figure 4 displays the variable importance plot using the OOB predictor. Factors 1 to 5 represent soil-related parameters, whereas factors 6 and 7 pertain to seismic parameters. In this figure, the OOB predictor is used to illustrate the importance of each factor, where a higher OOB predictor value indicates greater significance. The variable importance plot in the figure distinguishes between two groups of parameters using different color schemes: brown is used for factors 1 to 5, representing soil-related parameters, while blue is used for factors 6 and 7, representing seismic parameters.

According to the OOB predictor analysis, for soil-related parameters (factors 1 to 5), the OOB predictors for factors 1, 2, 3, and 5 are less than 1. However, the OOB predictor for factor 4 (SPT-N value) is 2.74, indicating that factor 4 (SPT-N value) holds the highest significance ranking among the soil-related parameters. As for seismic parameters, for factors 6 and 7, the OOB predictor for factor 6 is only 0.41, whereas the OOB predictor for factor 7 (peak ground acceleration) is 3.19, showing that factor 7 (peak ground acceleration) ranks the highest in significance among the seismic parameters.

According to the variable importance plot shown in Fig. 4, factor 4 (SPT-N value) and factor 7 (peak ground acceleration) are the most significant soil and seismic factors, respectively. This analysis reveals that factor 7 (peak ground acceleration) is the most critical factor overall, illustrating the strong connection between soil liquefaction and earthquake-induced ground acceleration. Additionally, factor 4 (SPT-N value), an inherent property of the soil, is a crucial parameter in assessing the potential for soil liquefaction.

It is crucial to consider that when assessing soil liquefaction potential, both soil characteristics and seismic factors that may trigger liquefaction need to be examined. The most crucial aspect is the soil properties at the site, as determined by field investigations, since these are the inherent conditions of the soil. Earthquakes with a certain minimum intensity act as the external triggers for soil liquefaction. Typically, soil properties can be acquired through preliminary field investigations, but the peak ground acceleration of an earthquake is an uncontrollable and unpredictable factor.

### Random forest structure

**Fig. 5:**
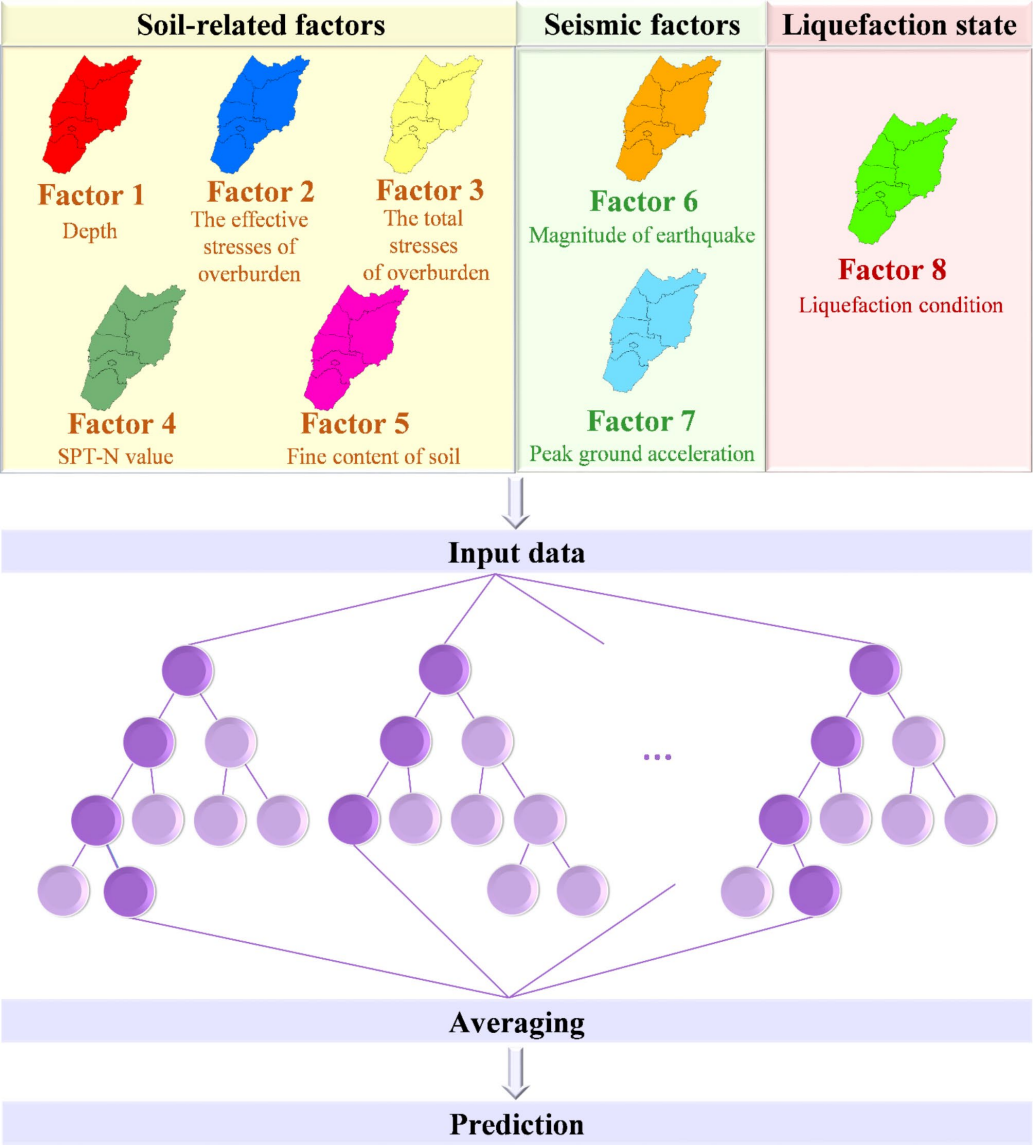
The structure of the random forest model.

The structure of a RF, as shown in Fig. 5, involves the creation of an ensemble of decision trees. Each decision tree is constructed independently using a random subset of the training data and a random subset of the input features. During the training process, each tree is grown to its maximum depth or until a specified stopping criterion is met, such as a minimum number of samples required to split a node or a maximum depth limit. Once all the decision trees are built, predictions are made by aggregating the predictions of each individual tree. For regression tasks, the final prediction is typically the mean of the predictions from all the trees, while for classification tasks, the final prediction is often determined by a majority vote among the trees. The incorporation of randomness in the construction of each tree assists in reducing correlations among the trees and mitigating the risk of overfitting. Assuming there is a database *D*, which can be represented as follows:

20 $${\text{(}}{x_i},{y_i}){\text{ for }}i=1,{\text{ }}2, \ldots ,{\text{ }}N{\text{ }}\& {\text{ }}{x_{i1}},{x_{i2}}, \ldots {\text{,}}{x_{ip}}.$$

In this equation, *x*, *y*, *N*, and *p* are the input, output, data number, and number of factors. If *D* is divided into *M* regions and ${D_1},{D_2}, \ldots {\text{,}}{D_M}$ is obtained, and a constant ${c_m}$ is used to represent the simulated output $f(x)$ of each region, the following equation can be obtained:

21 $$f(x)=\sum\limits_{{m=1}}^{M} {{c_m}I(x \in {D_m})} ,$$

where *I* is an indicator function. By incorporating the least squares sum as a criterion, the optimal constant, ${\hat {c}_m}$, can be obtained as the average of the output values, ${D_m}$, within the region:

22 $${\hat {c}_m}={\text{average }}({y_i}\left| {{x_i} \in {D_m})} \right.$$

Suppose there exists a categorical variable *j* and a split point *s*, and the database is divided into two databases, as shown in the following equation:

23 $${D_1}(j,s)=\left\{ {x\left| {{x_j} \leq s} \right.} \right\}{\text{ and }}{D_2}(j,s)=\left\{ {x\left| {{x_j}>s} \right.} \right\}.$$

According to the above equation, searching for the appropriate categorical variable *j* and split point *s* yields the following equation:

24 $$\mathop {\hbox{min} }\limits_{{j,s}} \left[ {\mathop {\hbox{min} }\limits_{{{c_1}}} \sum\limits_{{{x_i} \in {D_1}(j,s)}} {{{\left( {{y_i} - {c_1}} \right)}^2}} +\mathop {\hbox{min} }\limits_{{{c_2}}} \sum\limits_{{{x_i} \in {D_2}(j,s)}} {{{\left( {{y_i} - {c_2}} \right)}^2}} } \right].$$

Based on the equation above, the internal minimization for any combination of *j* and *s* can be derived from the following expression:

25 $${\hat {c}_1}={\text{average }}({y_i}\left| {{x_i} \in {D_1}(j,s))} \right.{\text{ \& }}{\hat {c}_2}={\text{average }}({y_i}\left| {{x_i} \in {D_2}(j,s))} \right.$$

By utilizing the above two equations, the optimal (*j*, *s*) pair can be obtained, leading to the division of the data into two regions. Repeating the aforementioned computations allows the data to be continuously split into all resultant regions. If a decision tree *T* divides the data into ${D_m}$ regions through *m* nodes, where ${N_m}$ denotes the total number of regions, ${\hat {c}_m}$ can be expressed as follows:

26 $${\hat {c}_m}=\frac{1}{{{N_m}}}\sum\limits_{{{x_i} \in {D_m}}} {{y_i}} .$$

Bagging, also known as bootstrap aggregation, is a method of obtaining an aggregated predictor by generating multiple versions of predictors and aggregating them. When this aggregated predictor is used to predict numerical results, it averages the results of each version and can also conduct a majority vote on the prediction results. Different versions of predictors are obtained by sampling from the dataset, with each sampling considered as modeling a new dataset. Assuming a database *D* as described earlier, it is divided into smaller datasets, ${\tilde {D}_b}$, and ${\text{ }}b=1,{\text{ }}2, \ldots ,{\text{ }}B$ to obtain ${\tilde {D}_1} , {\tilde {D}_2} , \ldots , {\tilde {D}_B}$. The sampling process involves a fixed number of samples each time, and the sampled data is replaced back into the original dataset before the next sampling. After calculating each small dataset, ${\tilde {D}_b}$, using the base algorithm, their results, $\tilde {f}(x)$, are collected, and the final training result is obtained by averaging all results, ${\tilde {f}_{bag}}(x)$, expressed as follows:

27 $${\tilde {f}_{bag}}(x)=\frac{1}{B}\sum\limits_{{b=1}}^{B} {{{\tilde {f}}_b}(x)} ,$$

where ${\tilde {f}_b}(x)$ is the output results obtained for each small dataset by the base algorithm. In a RF, each decision tree requires setting certain hyperparameters, such as the number of learning cycles, the learning rate, and the number of features used for node splitting, among others. The selection of these hyperparameters may affect the model’s performance. In this study, a cross-validation method named Leave-One-Out Cross Validation (LOOCV) was employed. The mathematical principle of LOOCV can be described simply as training and testing the dataset *n* times, where *n* is the number of samples in the dataset. During each test, only one sample is used, and the remaining $n - 1$ samples are used to train the model.

### Hyperparameter tuning for random forest

The hyperparameter tuning for the RF model involves performing convergence analysis of these hyperparameters and assessing the model’s reliability and stability using various performance metrics. The parameters for the RF model in this case are as follows: number of variables to sample is set to all, surrogate splits are set to on, pruning is set to off, the splitting criterion is mean square error (MSE), the decision tree type is regression, and the quadratic error tolerance is set to 10^− 6^.

**Fig. 6:**
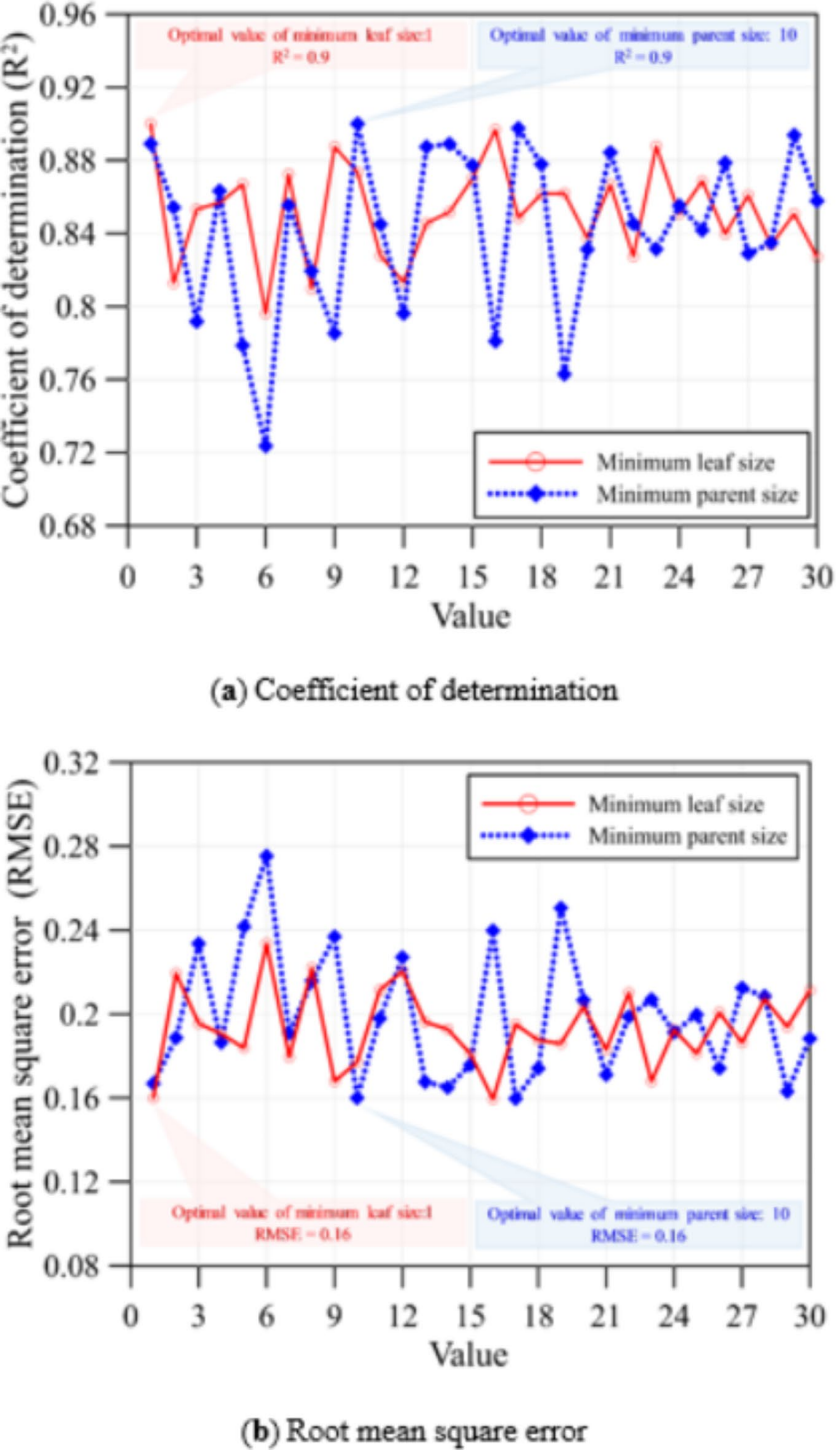
Convergence analysis for the minimum parent size and minimum leaf size in RF.

Figure 6 presents the convergence analysis for the minimum parent size and minimum leaf size in RF. This study examines the RMSE and R² for these parameters. The convergence analysis was conducted using various parameter values (ranging from 1 to 30) based on calculated error evaluation metrics. The results indicate that the optimal value for the minimum parent size is 10, and for the minimum leaf size, it is 1. Bayesian optimization was employed to optimize the number of ensemble learning cycles, learning rate, and maximum number of splits. The parameter analysis results are detailed in Table 3.

**Table 3:** The model parameters in the RF.

Number of variables to sample	All
Surrogate splits	On
Pruning	Off
Splitting criterion	MSE
Type	Regression
Quadratic error tolerance	10^− 6^
Minimum parent size	10
Minimum leaf size	1
Number of ensemble learning cycles	Bayesian optimization
Learning rate	Bayesian optimization
Maximum number of splits	Bayesian optimization

## Results

### Validation

The dataset consists of 540 data, with 70% (378 data) used for training and the remaining 30% (162 data) used for testing. The optimal model parameters are detailed in Table 3. The performance of the proposed RF predictive models indicates that the R^2^ for the training and testing datasets were 0.99 and 0.90, respectively. These findings suggest a strong correlation between past liquefaction occurrences and the model’s predictions. Table 4 illustrates the performance observed across 50 iterations for the training and testing dataset. These metrics validate the efficiency of the model employing seven factors as inputs.

**Table 4:** Performance across 50 iterations for both the training and testing datasets.

Performance indices	Ideal value	Training	Testing
R^2^	1	0.99	0.90
RMSE	0	7.54 × 10^–3^	1.60 × 10^–1^
VAF	100	99.98	89.21
PI	2	1.99	1.63
MAE	0	3.46 × 10^–3^	5.11 × 10^–2^
WI	1	0.99	0.97
WMAPE	0	7.10 × 10^–3^	9.62 × 10^–2^
NS	1	0.99	0.89
AIC	NA	–3681.0	–579.4

To further investigate the accuracy of the RF model for evaluating soil liquefaction, additional analyses were conducted on 270 liquefaction and 270 non-liquefaction cases. In comparing with other methods such as NCEER, HBF, and revised HBF, this study employed the trained model (based on the 70% data subset) to directly assess accuracy by inputting all 540 data. Therefore, this approach effectively incorporates all 540 cases for evaluation. The accuracy for these 540 data were calculated using the RF model developed in this study, as well as the NCEER, HBF, and revised HBF methods. Table 5 shows the accuracy for both liquefaction and non-liquefaction cases. The RF model achieved an accuracy of 99.63% for liquefaction cases and 98.15% for non-liquefaction cases, with an overall accuracy of 98.89% for all 540 data. In contrast, the overall accuracy using NCEER, HBF, and revised HBF were only 97.45%, 94.70%, and 94.70%, respectively. This demonstrates that the RF model developed in this study provides higher accuracy in evaluating soil liquefaction compared to the NCEER, HBF and revised HBF methods.

**Table 5:** Accuracy for of liquefaction and non-liquefaction cases.

Method		NCEER	HBF	Revised HBF [20]	This study
Accuracy (%)	Liquefaction occurred	100.00	94.44	96.30	99.63
	Liquefaction not occurred	94.56	94.98	92.89	98.15
	Overall	97.45	94.70	94.70	98.89

### Prediction

Traditional soil liquefaction assessment methods rely on a comprehensive analysis of soil and seismic parameters, including depth, effective and total stresses of overburden, SPT-N value, fine soil content, earthquake magnitude, peak ground acceleration, and liquefaction occurrence, totaling seven factors. However, in practice, it may not always be possible to obtain all parameters due to limitations in time, budget, or availability of data. Therefore, this study further explores whether the RF model can still perform soil liquefaction potential assessment when input soil and seismic parameters are incomplete.

The dataset considered in this study still comprises 540 data points, but the analysis accounts for missing input factors within these points. Overall, this study involves 16 cases: case 1 has no missing factors, utilizing all seven factors as input variables. Cases 2 to 6 each lack one factor, while cases 7 to 16 each lack two factors. Table 6 summarizes the missing factors for each case.

It is important to note that seismic conditions are crucial factors for analysis when evaluating the potential for soil liquefaction at a site. These include earthquake magnitude (factor 6) and peak ground acceleration (factor 7), which directly impact seismic forces and propagation characteristics at the site. Since soil liquefaction is caused by seismic activity, it is vital to incorporate seismic conditions as essential input factors to assess the likelihood of soil liquefaction during an earthquake. Therefore, in this case, only soil factors are regarded as potential missing factors, while earthquake magnitude (factor 6) and peak ground acceleration (factor 7) are identified as essential input factors.

**Table 6:** Description of missing factors for each case.

Case	Missing data	Case	Missing data
1	None	9	Factor 1 and Factor 4
2	Factor 1	10	Factor 1 and Factor 5
3	Factor 2	11	Factor 2 and Factor 3
4	Factor 3	12	Factor 2 and Factor 4
5	Factor 4	13	Factor 2 and Factor 5
6	Factor 5	14	Factor 3 and Factor 4
7	Factor 1 and Factor 2	15	Factor 3 and Factor 5
8	Factor 1 and Factor 3	16	Factor 4 and Factor 5

Factor 1 is the depth; factor 2 is the effective stresses of overburden; factor 3 is the total stresses of overburden; factor 4 is the SPT-N value; factor 5 is the fine content of soil.

The analysis continues using the previously established optimal RF model. The input factors for the RF model are set according to the scenarios described in these 16 cases, with the output being the soil liquefaction status. The study uses the RF model to predict the soil liquefaction status for these 16 scenarios and compares the results with actual soil liquefaction conditions.

Initially, this study calculates the error metrics for the testing data across the 16 cases using the RF model. Table 7 lists the results of the nine error metrics for the testing data across the 16 cases. For example, in instances where one factor is absent (cases 2 to 6), all five cases exhibit an R² of 0.8 or higher, indicating commendable performance. Similarly, in scenarios where two factors are missing (cases 7 to 16), all ten cases demonstrate an R² exceeding 0.7, affirming the RF model’s reliability in analyzing these 16 cases.

Subsequently, this study calculates scores for these 16 scenarios based on nine error metrics: R², NS, VAF, RMSE, WI, AIC, WMAPE, PI, and MAE. The calculation method involves evaluating each of the nine error metrics for the 16 cases. Based on the results of these metrics across all cases, as detailed in Table 7, this study ranked and assigned scores to each case. Scores ranged from 16 points for the top-performing case (such as case 1, achieving the highest R^2^ score) to 1 point for the least-performing case (case 16). The remaining cases were scored in descending order according to their respective error metric results. Each case was evaluated across all nine error metrics, and these scores were summed to derive a total sum.

**Table 7:** Results of the nine error metrics across 16 cases.

Case	*R* ^2^	RMSE	VAF	PI	MAE	WI	WMAPE	NS	AIC
1	0.90	1.60 × 10^–1^	89.21	1.63	5.11 × 10^–2^	0.97	9.62 × 10^–2^	0.89	-579.4
2	0.88	1.74 × 10^–1^	87.04	1.57	8.00 × 10^–2^	0.97	1.73 × 10^–1^	0.87	-554.2
3	0.86	1.90 × 10^–1^	84.03	1.5	6.42 × 10^–2^	0.96	1.39 × 10^–1^	0.84	-525.9
4	0.88	1.74 × 10^–1^	87.7	1.58	8.91 × 10^–2^	0.97	1.90 × 10^–1^	0.88	-554.8
5	0.80	2.30 × 10^–1^	76.85	1.33	9.94 × 10^–2^	0.94	2.15 × 10^–1^	0.76	-464.4
6	0.86	1.91 × 10^–1^	84.81	1.51	5.26 × 10^–2^	0.96	1.12 × 10^–1^	0.85	-525
7	0.76	2.55 × 10^–1^	73.84	1.24	7.27 × 10^–2^	0.93	1.44 × 10^–1^	0.73	-433.1
8	0.87	1.76 × 10^–1^	87.29	1.57	9.40 × 10^–2^	0.97	2.00 × 10^–1^	0.87	-553.7
9	0.77	2.69 × 10^–1^	76.44	1.26	1.38 × 10^–1^	0.93	2.97 × 10^–1^	0.75	-415.9
10	0.78	2.44 × 10^–1^	75.85	1.29	7.08 × 10^–2^	0.94	1.40 × 10^–1^	0.75	-447.6
11	0.76	2.55 × 10^–1^	72.73	1.22	8.43 × 10^–2^	0.93	1.67 × 10^–1^	0.72	-432.8
12	0.77	2.43 × 10^–1^	74.73	1.27	8.82 × 10^–2^	0.94	1.91 × 10^–1^	0.75	-448.8
13	0.77	2.46 × 10^–1^	73.93	1.26	8.59 × 10^–2^	0.94	1.70 × 10^–1^	0.73	-444.9
14	0.77	2.41 × 10^–1^	74.65	1.27	1.05 × 10^–1^	0.94	2.23 × 10^–1^	0.75	-451
15	0.74	2.59 × 10^–1^	70.66	1.18	1.02 × 10^–1^	0.93	2.21 × 10^–1^	0.7	-428
16	0.70	2.85 × 10^–1^	66.71	1.07	1.99 × 10^–1^	0.91	4.31 × 10^–1^	0.66	-396.9

In Case 1, the maximum total sum (144) was obtained. To facilitate comparison with accuracy, this sum was converted into a score using the following formula: score is equal to the sum divided by the maximum total sum across 16 cases (144 in case 1), then multiplied by 100. This transformation aids in comparing and presenting scores relative to different standards, making comparisons more intuitive. The scores for the nine error metrics across the 16 cases are summarized in Table 8.

Finally, the scores for the nine error metrics across the 16 cases are obtained. Precision assessment is conducted for each metric, with the case achieving the highest precision for each metric receiving the top score. Finally, the scores are aggregated, with the maximum attainable score being 100.

**Table 8:** Score of the nine error metrics across 16 cases.

Case	*R* ^2^	RMSE	VAF	PI	MAE	WI	WMAPE	NS	AIC	Sum	Score
1	16	16	16	16	16	16	16	16	16	144	100.00
2	15	15	13	13	11	15	9	14	14	119	82.64
3	12	12	11	11	14	11	14	11	12	108	75.00
4	14	14	15	15	7	14	8	15	15	117	81.25
5	10	10	10	10	5	7	5	10	10	77	53.47
6	11	11	12	12	15	12	15	12	11	111	77.08
7	4	5	4	4	12	5	12	5	5	56	38.89
8	13	13	14	14	6	13	6	13	13	105	72.92
9	8	2	9	6	2	2	2	6	2	39	27.08
10	9	7	8	9	13	10	13	9	7	85	59.03
11	3	4	3	3	10	4	11	3	4	45	31.25
12	7	8	7	8	8	8	7	8	8	69	47.92
13	6	6	5	5	9	9	10	4	6	60	41.67
14	5	9	6	7	3	6	3	7	9	55	38.19
15	2	3	2	2	4	3	4	2	3	25	17.36
16	1	1	1	1	1	1	1	1	1	9	6.25

**Fig. 7:**
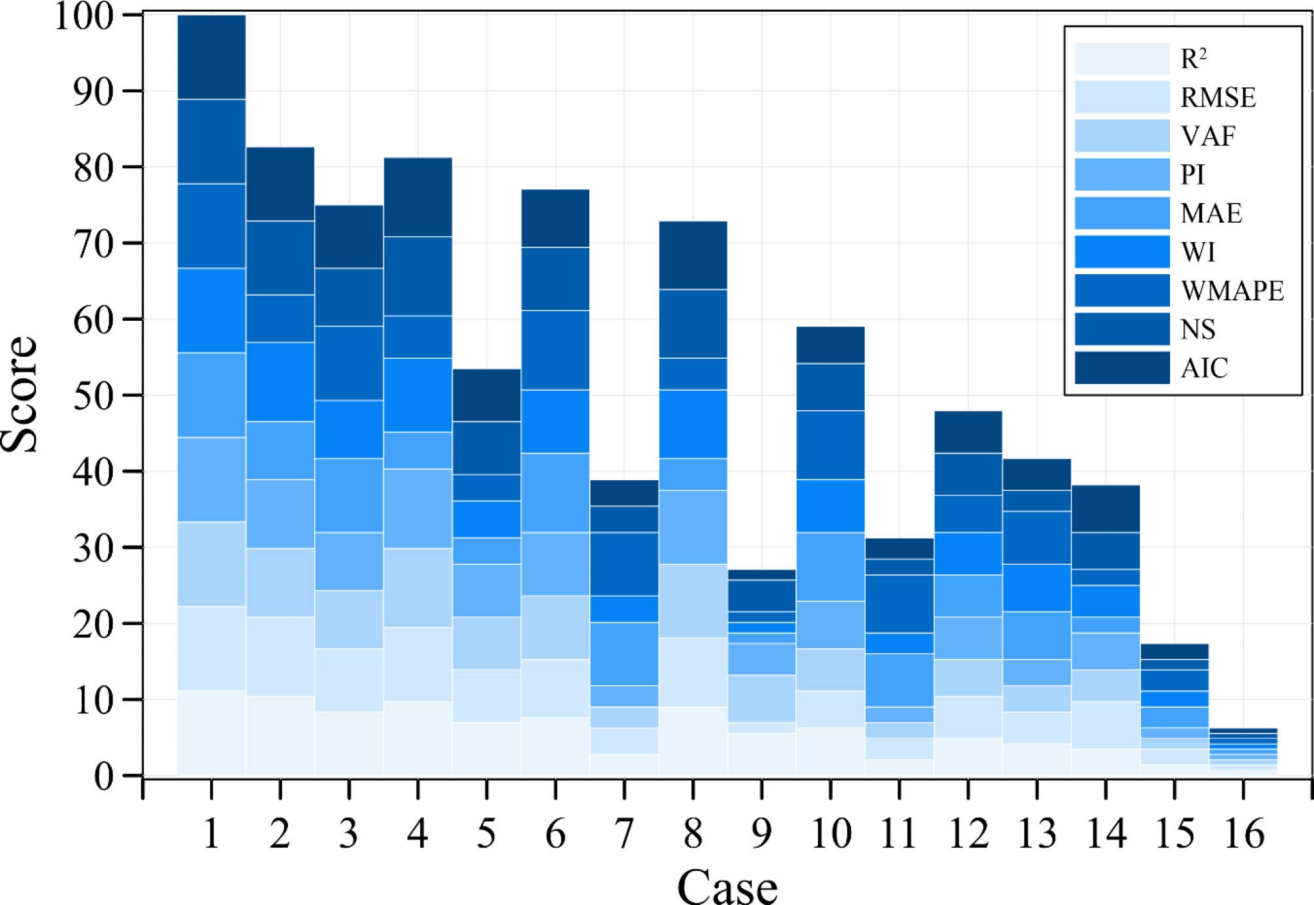
Results of the score for 16 cases.

Figure 7 presents the score results for the 16 cases. Since case 1 includes all seven factors as input variables, the RF analysis results for soil liquefaction are the most accurate, achieving a score of 100. For scenarios where one factor is missing, such as cases 2 to 6, the analysis shows that all cases, except case 5, scored above 75. Case 5, which lacks factor 4 (SPT-N value), only scored 53.47.

The finding is consistent with the previous OOB predictor importance analysis, which identified factor 4 (SPT-N value) as the most crucial input. Therefore, the absence of factor 4 (SPT-N value) severely impacts the soil liquefaction analysis, resulting in inaccurate predictions. This trend is also seen in cases 7 to 14, where each scenario involves the absence of two factors. Specifically, in cases 9, 12, 14, and 16, where factor 4 is missing, the scores are all below 48. On the other hand, cases 11 and 15, which include factor 4, still only scored 31.25 and 17.36, respectively. In cases 11, factor 2 (effective stresses of overburden) and factor 3 (total stresses of overburden) are absent, whereas in cases 15, factor 3 (total stresses of overburden) and factor 5 (fine content of soil) are missing. The results demonstrate that the absence of these two factors in the input leads to lower scores. However, it is important to emphasize that the score used in this study serves only as a reference error indicator for evaluating the RF model’s performance. The validation of the RF model’s analysis results should still rely on accuracy.

Table 9 provides a summary of the score and accuracy for both liquefaction and non-liquefaction cases. In scenarios where one factor is missing, such as cases 2 to 6, results demonstrate that the overall accuracy for both liquefaction and non-liquefaction cases exceed 96%. Similarly, in cases with two missing factors, such as in cases 7 to 16, all achieve an accuracy of over 95%. Moreover, according to this study’s accuracy results, in cases 9, 12, 14, and 16, where factor 4 (SPT-N value) is missing, the accuracy is indeed lower than in other cases. Nonetheless, even without factor 4, these cases still achieve an accuracy of over 95%.

**Table 9:** Prediction results for of liquefaction and non-liquefaction cases.

Case	1	2	3	4	5	6	7	8
Score	100	82.64	75	81.25	53.47	77.08	38.89	72.92
Accuracy (%)	98.89	98.34	97.60	98.71	96.67	98.52	96.86	97.63

Case	9	10	11	12	13	14	15	16
Score	27.08	59.03	31.25	47.92	41.67	38.19	17.36	6.25
Accuracy (%)	95.75	98.34	96.67	95.75	97.60	95.75	96.67	95.19

**Fig. 8:**
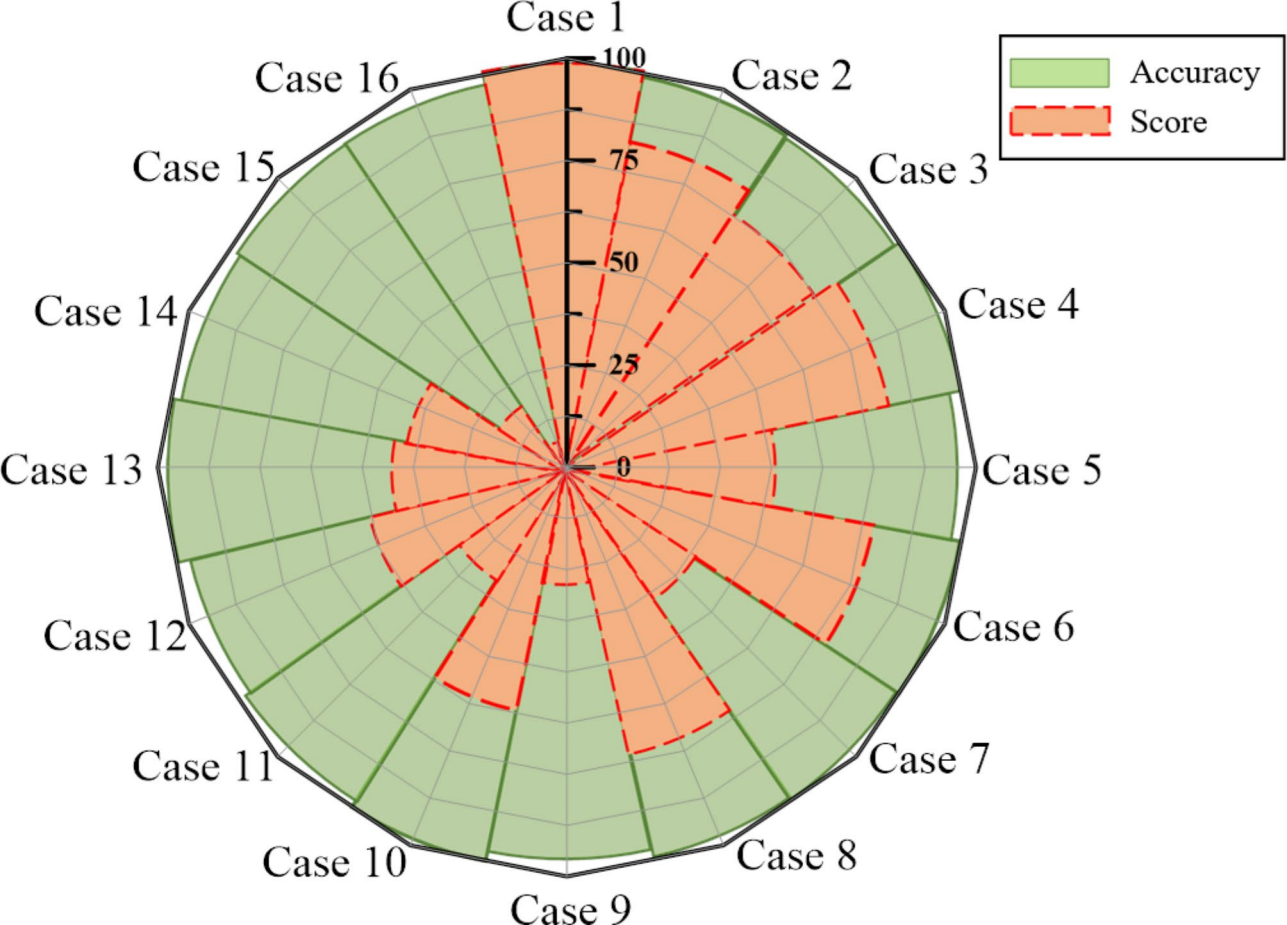
Comparing the scores and accuracy for 16 cases.

Further comparison of the scores and accuracy for the 16 cases is depicted in Fig. 8. These findings underscore the effectiveness of the proposed RF model, which has demonstrated its capability to accurately predict soil liquefaction events even in situations with limited input factors—an accomplishment that conventional methods often struggle to achieve.

In this study, in addition to using random forest (RF), other machine learning methods were employed for model development, including deep neural networks (DNN), support vector machines (SVM), and long short-term memory networks (LSTM). The accuracy of the models was calculated to compare the different machine learning methods, and the analysis results are presented in the table below.

The dataset used in this study remains at 540 data, but the analysis accounts for missing input factors within these points. The study evaluates 16 different cases: Case 1 includes all seven input factors with none missing, while Cases 2 through 6 each omit one factor, and Cases 7 through 16 omit two factors. Table 6 outlines the missing factors for each case.

Table 10 presents the results for nine error metrics on the testing data across the 16 cases, comparing various machine learning methods. When all seven input factors are used, LSTM demonstrates the highest accuracy at 99.07%, followed by RF with 98.89%, and DNN and SVM with accuracies of 96.85% and 95.74%, respectively.

This study also explores how the accuracy of each machine learning model in predicting soil liquefaction is impacted by a reduction in input factors. Results indicate that RF consistently maintains an accuracy of at least 95% across all scenarios from Case 2 to Case 16, whereas LSTM shows less stability. Similar trends are observed with the other methods.

This study highlights the advantage of the RF model in evaluating and predicting soil liquefaction, particularly in scenarios with missing input factors. RF is able to achieve over 95% prediction accuracy even when some input factors are absent.

**Table 10:** Comparison of accuracy for different machine learning methods across the 16 cases.

Case		1	2	3	4	5	6	7	8
Accuracy (%)	DNN	96.85	96.67	95.19	96.30	90.37	95.00	93.15	93.70
	RF	98.89	98.34	97.60	98.71	96.67	98.52	96.86	97.63
	SVM	95.74	95.74	92.59	96.11	90.37	95.74	90.37	95.19
	LSTM	99.07	98.52	98.52	73.52	92.96	97.41	95.74	70.89

Case		9	10	11	12	13	14	15	16
Accuracy (%)	DNN	90.19	94.44	93.89	89.63	94.63	89.81	94.07	89.63
	RF	95.75	98.34	96.67	95.19	97.60	95.75	96.67	95.19
	SVM	82.96	95.19	92.59	87.04	90.37	88.15	95.00	90.37
	LSTM	90.37	97.41	72.56	90.19	91.30	73.52	70.89	89.63

## Conclusion

A reliable predictive RF model based on soil and seismic parameters is developed for predicting liquefaction potential in central Taiwan. The primary discoveries of this investigation can be outlined as follows:

1. This study begins by examining the importance of input factors in soil liquefaction using OOB predictor importance analysis. The results demonstrate that SPT-N value and peak ground acceleration rank highest in importance, indicating their significant influence on liquefaction. This emphasizes the essential role of these factors as fundamental parameters for liquefaction analysis.
2. Using a broad dataset containing 540 entries from various sources, including SPT, soil properties, seismic activity indicators, and past liquefaction occurrences. Through cross-validation and comparison with observed liquefaction events, the RF model’s performance is thoroughly evaluated. The results demonstrate R^2^ of 0.99 for the training dataset and 0.90 for the testing dataset, respectively. These findings illustrate a robust correlation between historical liquefaction events and the model’s predictive accuracy.
3. Moreover, the proposed RF model exhibits the capability to precisely predict soil liquefaction events even in situations where input factors are lacking. In instances where one factor is absent, the research findings indicate that the overall accuracy exceed 96%. For cases with two missing factors, nearly all achieve an accuracy of over 95%. When comprehensive parameters are not available during on-site evaluations, conventional liquefaction analysis cannot be applied to assess soil liquefaction potential. This underscores the superiority of the proposed RF model.
4. Findings in this study emphasize the model’s capability to accurately forecast liquefaction potential with a notable level of reliability and precision. This research not only advances predictive modeling techniques in geotechnical engineering but also bears significant implications for enhancing hazard assessment and risk management strategies in seismic-prone regions of Taiwan.

## Data Availability

Sequence data that support the findings of this study have been found and access in the appendix I of the paper, “Hwang, J. H., Khoshnevisan, S., Juang, C. H., & Lu, C. C. (2021). Soil liquefaction potential evaluation–An update of the HBF method focusing on research and practice in Taiwan. Engineering Geology, 280, 105926.” https://doi.org/10.1016/j.enggeo.2020.105926.
